# Plasma protein biomarkers of *Plasmodium falciparum* infection in pregnant women: a high-throughput proteomics study

**DOI:** 10.3389/fcimb.2025.1594088

**Published:** 2025-07-08

**Authors:** Bernard N. Kanoi, Harrison Waweru, Francis M. Kobia, Joseph Mukala, Peter Kirira, Dominic Mogere, Radiosa Gallini, Mikael Åberg, Manu Vatish, Jesse Gitaka, Masood Kamali-Moghaddam

**Affiliations:** ^1^ Centre for Malaria Elimination, Institute of Tropical Medicine, Mount Kenya University, Thika, Kenya; ^2^ School of Public Health, Mount Kenya University, Thika, Kenya; ^3^ School of Pure and Applied Sciences, Mount Kenya University, Thika, Kenya; ^4^ Department of Immunology, Genetics and Pathology, Science for Life Laboratory, Uppsala University, Uppsala, Sweden; ^5^ Department of Medical Sciences, Clinical Chemistry and SciLifeLab Affinity Proteomics, Uppsala University, Uppsala, Sweden; ^6^ Nuffield Department of Women’s and Reproductive Health, University of Oxford, Oxford, United Kingdom

**Keywords:** pregnancy-associated malaria, malaria in pregnancy, biomarkers, *Plasmodium falciparum*, proteomics, proximity extension assay (PEA)

## Abstract

**Introduction:**

Pregnant women in sub-Saharan Africa face heightened susceptibility to *Plasmodium falciparum* malaria, with placental sequestration driving adverse outcomes. The infection may lead to pregnancy-associated malaria (PAM) because of the sequestration of *Plasmodium falciparum*-infected erythrocytes in the placental intervillous space. Although there are several tools for diagnosing malaria infection during pregnancy, including blood smear microscopic examination, rapid diagnostic tests, and PCR, there are no tools for detecting placental infection and, by extension, any dysfunction associated with PAM. Thus, PAM, specifically placental infection, can only be confirmed via postnatal placental histopathology. Therefore, there is an urgent need for specific plasma biomarkers of PAM.

**Methods:**

Here, we used the high throughput proximity extension assay to screen plasma from malaria-exposed pregnant women for differentially expressed proteins that may serve as candidate biomarkers of *Plasmodium falciparum* infection during pregnancy, with future potential to inform diagnosis of PAM or adverse malaria outcomes. Such biomarkers may also elucidate the pathophysiology of PAM.

**Results:**

Using proximity extension assay (PEA), we identified elevated IgG Fc receptor IIb (FCGR2B) and heme oxygenase-1 (HO-1) in malaria-positive pregnancies, while neurturin (NRTN) and IL-20 were downregulated.

**Discussion:**

IL-20 emerged as a top candidate biomarker, warranting validation in large cohorts with placental histopathology.

## Introduction

Placental sequestration of P. falciparum-infected erythrocytes defines pregnancy-associated malaria (PAM), contributing to approximately100,000 neonatal deaths annually in endemic regions (Rogerson et al., 2007). Despite the availability of numerous diagnostic tools for detecting malaria during pregnancy, there is a lack of a definitive test for determining actual placental infection and dysfunction within the context of PAM (Brabin, 1983). PAM has several similarities with pre-eclampsia, for which several biomarkers of placental stress and dysfunction have been identified (Roberts and Lain, 2002; Brabin and Johnson, 2005). While some of these biomarkers are inflammatory markers that lack specificity, others, such as short-endoglin (s-endoglin) and soluble fms-like tyrosine kinase 1 (sFlt1), which are elevated in peripheral blood during pre-eclampsia, appear more specific to placental dysfunction (Jena et al., 2020). The predictive value of such biomarkers should be evaluated in the context of PAM to determine their effectiveness as indicators of PAM-associated placental infection and dysfunction (Gueneuc et al., 2017). This is crucial for enhancing the diagnosis of typically sub-microscopic malaria infections and malaria-induced placental dysfunction, thereby facilitating timely intervention in pregnant women. Indeed, comprehension of disease biology and the interaction between the malaria parasite and the human host is crucial for developing diagnostics, prognostics, and uncovering predictive biomarkers for PAM (Acharya et al., 2017). Elucidating the expression patterns of plasma proteins can provide insights into how PAM affects placental, maternal, or fetal wellbeing (Unger et al., 2015; Gueneuc et al., 2017).

Previous studies have investigated biomarkers specifically associated with *P. falciparum* infection during pregnancy. For instance, Boström et al. (2012) reported elevated levels of inflammatory cytokines, including IL-10 and TNF-α, in the plasma from women with placental malaria (Boström et al., 2012). Ruizendaal et al. (2015) reported increased expression of angiogenic markers such as sFlt-1 and s-Endoglin, particularly in women with placental infection (Ruizendaal et al., 2017). Complement activation has also emerged as a key feature of PAM, as demonstrated in studies by Hountohotegbe et al. (2020) and Santiago et al (Santiago et al., 2023). Furthermore, Megnekou et al. (2015) showed distinct cytokine profiles in women with PAM, supporting the involvement of immune modulation in disease pathophysiology (Megnekou et al., 2015). These studies collectively underscore the multifactorial nature of host immune responses in PAM and support the current need for discovery and validation of plasma-based biomarkers for early detection and management of malaria in pregnancy.

The rapid development of proteomics platforms, such as multiplex proximity extension assay (PEA), offers highly effective, high-throughput strategies for studying malaria-associated molecular pathophysiological changes (Wik et al., 2021). PEA technology enables simultaneous measurement of a large number of proteins using minute amount of sample (Acharya et al., 2017; Dayon et al., 2022). Studies using this approach have demonstrated its clinical utility in identifying important biomarkers for various diseases, including cancer, post COVID-19, ischemic stroke, and early pregnancy bleeding (Assarsson et al., 2014; Dayon et al., 2022; Allan-Blitz et al., 2023; Angerfors et al., 2023; Davies et al., 2023; Guterstam et al., 2023). Although the long-term goal of such biomarker studies is to identify predictors of placental dysfunction or adverse pregnancy outcomes, this study focuses on the discovery phase—namely, the identification of differentially expressed proteins in the plasma of *P. falciparum*-infected *vs.* non-infected pregnant women. In this study, we used PEA to screen plasma from malaria-exposed pregnant women to identify differentially expressed proteins. Using this strategy, we identified four proteins significantly differentially expressed in samples from malaria-infected pregnant women compared with non-infected pregnant counterparts, suggesting that their alteration is *P. falciparum-*driven. Such proteins can potentially develop into biomarkers of PAM or adverse malaria outcomes.

## Material and methods

### Study design & population

This study used plasma samples collected from a longitudinal cohort of pregnant women undergoing routine antenatal care at Webuye County Hospital, Bungoma County, western Kenya between 2020 and 2021. The region is characterized by high malaria transmission (Platt et al., 2018). The study protocol and the use of this well-characterized biobank of samples were approved by the Independent Ethics Research Committee (IERC) of Mount Kenya University (No# MKU/IERC/0543, MKU/IERC/2461). The study protocol complied with the International Conference on Harmonization Good Clinical Practices and the Declaration of Helsinki guidelines (Dixon, 1998). Briefly, the enrolled cohort consisted of women undergoing routine intermittent preventive malaria treatment in pregnancy (IPTp) with sulfadoxine-pyrimethamine (IPTp-SP) (Peters et al., 2007). The participants were followed up approximately every 4 weeks until delivery. All study participants gave written informed consent before joining the study. Plasma samples were collected 4 weeks after IPTp administration and frozen immediately, and then transported on dry ice to the Centre for Malaria Elimination laboratories at Mount Kenya University and immediately stored in a -80°C deep freezer without cold-chain interruption. Each plasma sample was matched with corresponding anonymized demographic and pregnancy outcome data. Data on pregnancy progress and outcomes were collected during the scheduled and unscheduled visits. Malaria diagnosis was performed using both microscopy and a ParaCheck Rapid Diagnostic Kit (Orchid Biomedical Systems, India). Randomly selected participants were analyzed in the current study.

### Protein abundance measurement by multiplex proximity extension assay

Multiplex PEA technology was used to analyze protein expression in the plasma samples using the Olink Inflammation and Cardiovascular II protein panels (Olink™ Proteomics, Uppsala Sweden; https://olink.com), loading 1µL sample/panel. These panels were selected since they target inflammatory and vascular pathophysiological proteomic signatures, which were hypothesized to be altered by PAM. In PEA, each target protein is recognized by two antibodies attached to single-stranded DNA oligonucleotide (Assarsson et al., 2014). When a pair of antibodies binds to the same target protein, the conjugated DNA oligonucleotides are brought in proximity and hybridize to each other. Subsequently, the DNA molecules are extended to form a double-stranded DNA as template for quantitative PCR amplification (Assarsson et al., 2014). The results are presented as normalized protein expression (NPX) values, which are arbitrary units on a log2 scale, where a one-unit increase in NPX corresponds to a two-fold increase in protein concentration. The limit of detection (LOD) for each protein was determined based on three times the standard deviation (SD) above the NPX value of the negative controls in each run. In this study, a total of 183 different proteins were measured, where each panel included 92 proteins involved in diverse biological processes and 4 technical controls. Analyses were performed at the Uppsala Affinity Proteomics unit at SciLifeLab, Uppsala University.

### Data analysis

Data analyses were performed using R software (www.r-project.org). NPX values were used for analysis because they tend to follow a normal distribution. Samples from malaria infected group and the controls were randomized within the plates to minimize variability between plates. The inter-plate variation was further normalized for each protein in each plate by adding a z-score factor, which was calculated as follows: factor = (actual value - median of all samples)/standard deviation. To compare malaria cases and controls, NPX values were adjusted if a significant effect (adjusted p value < 0.05) for age on protein levels was found by linear regression in both the control and malaria groups.

For univariate analysis, the difference in protein levels between the malaria positive and malaria negative groups was examined using linear regression, using age and gravida as covariates. The dependent variable was NPX, and the independent variables were malaria status. The likelihood ratio test was used to assess the significance of the difference. The statistical significance of the differences between two groups was determined for continuous variables using the Mann–Whitney–Wilcoxon (MW test). For categorical variables, the Chi-square or Fisher’s exact test was used. The analysis of variance (ANOVA) test was used to compare more than two groups. The paired Mann–Whitney–Wilcoxon test was used to compare different malaria groups. Spearman’s rank rho was used to test the correlation coefficients or collinearity between each protein marker and other continuous variables. The Bonferroni–Dunn *post hoc* test was used to correct for multiple testing by adjusting the p-values for false discovery rate. A difference was considered significant if the q-value (adjusted p-value) was less than 0.05.

Additionally, elastic-net penalized logistic regression (ENLR) was used to identify a combination of analytes that could improve the discrimination between cases and controls. ENLR applies a penalty to the regression coefficients and finds groups of correlated variables. The optimal penalization proportion α was determined using grid search with a 10-fold cross-validation. The optimal tuning parameter λ was determined as the mean value of 100 iterative lambda values that minimized the model’s deviance. The regression coefficients were used to assess the contribution of individual proteins to the case-control discrimination. The ENLR model was estimated using the R package glmnet (Fredriksson et al., 2002). The regression coefficients for the selected proteins were then calculated by rerunning ENLR with only proteins with non-zero coefficients after 10 repetitions. To determine the number of proteins required to predict PAM, ROC curves were generated while adding one more protein at a time and then compared with the first ROC curve. This process was repeated until none of the ROC curves showed significant improvement.

## Results

### Protein reactivity

PEA is optimal for measuring of the levels of soluble plasma proteins in small sample volumes. Here, to identify proteins that might indicate *P. falciparum* infection during pregnancy, we probed the Olink Target 96 Inflammation and Olink Target 96 Cardiovascular II Panels (**Supplementary Table S1**) using plasma samples collected from 50 women, who were sampled at three different timepoints during pregnancy (**Table 1**). The initial sampling was performed at 12–18 weeks of gestation and two more samples were collected between 36–38 weeks of gestation. Twelve (12) women (median age: 24 years, range: 20–29 years) had malaria infection that was detectable using a rapid diagnostic test. The women in the different groups did not differ significantly in terms of age, gravidity, or hemoglobin levels, but they did differ in the gestational age (p<0.05).

**Table 1:** Characteristics of the women assessed in this study.

Characteristics	Malaria	No Malaria	Overall	P value
	(n=12)	(n=38)	(n=50)	
Age in years at enrollment, mean (range) years	24.0 (20.0 - 29.0)	27.3 (19.0 - 42.0)	26.9 (19.0 - 42.0)	0.778
Gestation in weeks, mean (range)^α#^	18.7 (10.0 - 26.0)	14.2 (4.0 - 28.0)	21.8 (4.0 - 28.0)	0.005
Gravida, mean (range)	1.8 (1.0 - 3.0)	2.5 (1.0 - 5.0)	2.3 (1.0 - 5.0)	0.070
Hemoglobin in g/dL, mean (IQR)^α^	11.5 (7.6 -13.6)	12.8 (9.6 - 15.8)	12.5 (7.6 -15.8)	0.011
Random sugar levels in mmol/L, mean (IQR)	5.4 (4.7 - 6.4)	4.9 (4.0 - 6.5)	5.0 (4.0-6.5)	0.100

^α^Statistically significant between the groups (P<0.05 Mann-Whitney-Wilcoxon test; followed by Bonferroni–Dunn post hoc test); Malaria was defined as any parasitemia with or without fever; IQR, interquartile range; ^#^Gestation (mean, range) in weeks refers to the gestational age at enrollment; All participants were of the same ethnicity.

### Protein reactivity and the characteristic of the sampled women

Protein reactivity to the proteomic panels was measured at three different visits per participant. The pattern of expression profiles varied considerably among the volunteers, with most proteins being present at different time points (first, second and third trimester) for most women. However, in about 10% of the women, less than 20 proteins were detected (**Supplementary Table S2**).

To assess if there was any relationship between plasma protein levels and participant characteristics, such as age, gravida, and gestational age, we conducted a correlation analysis to each of these parameters against the NPX for each protein. Although there was no correlation between protein levels and participants’ age, overall plasma protein levels were higher in samples from primigravida (first-time pregnant) women when compared with multigravida women (who have had 2–5 pregnancies), although the difference did not reach statistical significance (**Supplementary Table S2**).

We further assessed the relationship between markers of morbidity, including hemoglobin and random blood sugars, and expressed plasma proteins. We observed that 19 proteins were significantly negatively correlated with hemoglobin levels, and 9 proteins with random blood sugar levels (unadjusted P < 0.05). However, while initial correlations suggested potential associations between certain plasma proteins and clinical indicators such as hemoglobin and blood glucose levels, these associations did not remain statistically significant after correction for multiple testing False Discovery Rate adjustment. Therefore, no definitive conclusions can be drawn regarding their biological relevance at this stage, and any potential role in malaria-related or pregnancy-associated complications warrants further investigation in larger studies.

### Differential protein expression in malaria *vs*. no malaria women

**Figure 1:**
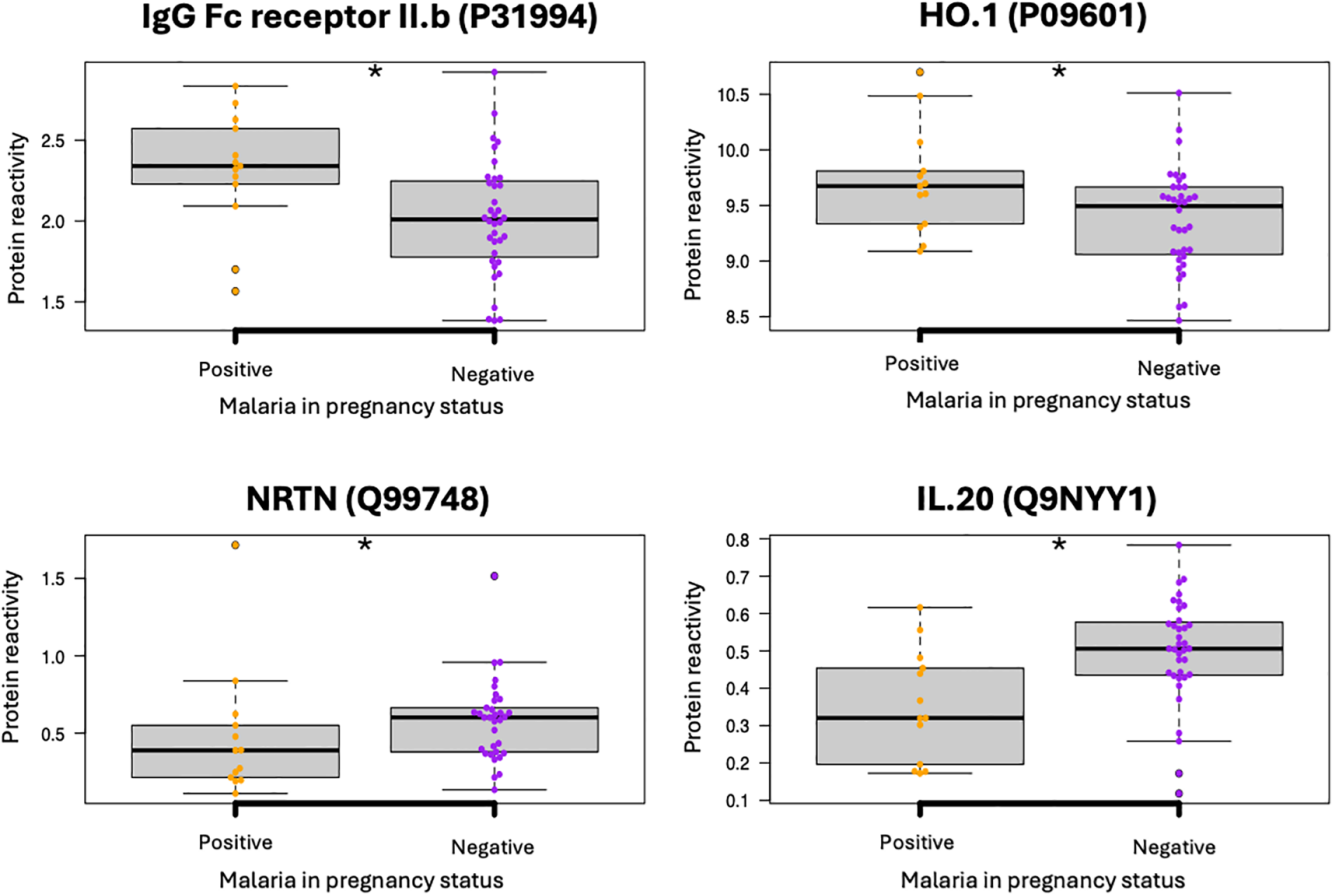
Differential protein expression in malaria positive *vs.* malaria negative women. The title represents protein Uniprot acronym and ID. Relative PEA counts, represented as protein expression levels that are derived from reactivity NPX/LOD, to allow protein-to-protein comparison. *Represent statistical difference between malaria positive and negative groups (Kruskal Wallis, P < 0.05).

Next, we sought to identify differential protein expression in malaria positive *vs.* malaria negative women. This analysis showed that 48 proteins were significantly differentially expressed in malaria positive *vs.* malaria negative women, in at least one of the three clinic visits (**Supplementary Table S2**). Notably, IgG Fc receptor IIb (FCGR2B; Uniprot ID: P31994) and heme oxygenase-1 (HO-1; Uniprot ID: P09601) were consistently highly expressed in malaria-positive samples when compared with malaria-negative samples (MW test, p = 0.008 and p = 0.014, respectively), while neurturin (NRTN; Uniprot ID: Q99748) and Interleukin 20 (IL-20; Uniprot: Q9NYY1) were consistently highly expressed in plasma from malaria-negative women (**Figure 1**) (p = 0.021 and p = 0.004, respectively). This data suggest that malaria infection may downregulate or upregulate various proteins.

**Figure 2:**
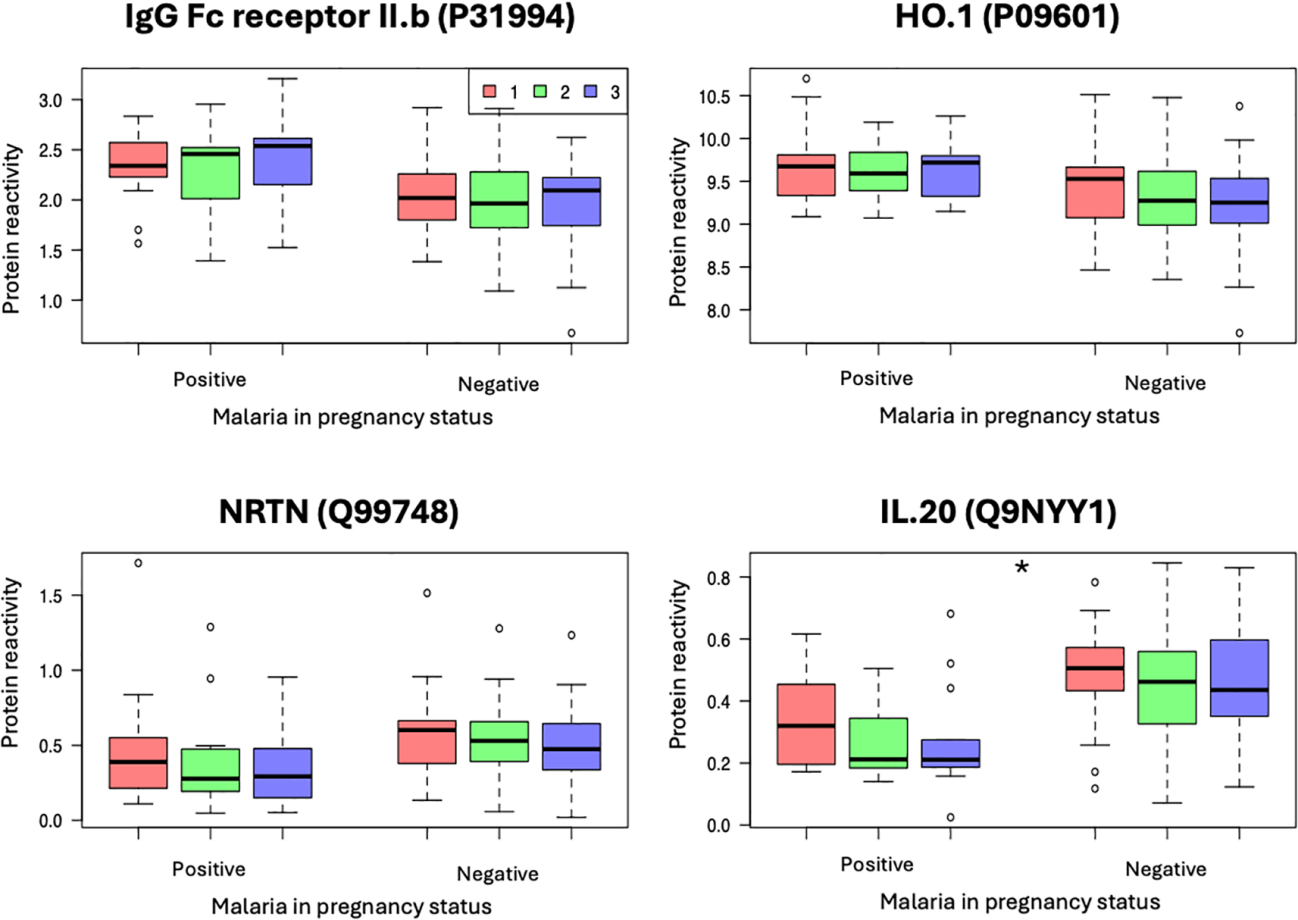
Kinetics of protein expression in malaria positive *vs.* malaria negative women at three time points during the pregnancy – 1^st^ (red), 2^nd^ (green) and 3^rd^ Visit (blue). *Represent statistical difference between hospital visits, (Kruskal Wallis, P < 0.05). Relative PEA counts, represented as protein expression levels.

We therefore sought to assess if the levels of these proteins fluctuated during the antepartum period using samples collected at different pregnancy timepoints. Except for IL-20, whose plasma levels reduced during the follow-up period (Kruskal Wallis, p < 0.05), there was no significant change in the other proteins, suggesting that the initial induction by malaria infection did not alter the levels of these proteins during the follow-up period. The top differentially expressed proteins are presented in **Figure 2**, and the full protein list is presented in **Supplementary Tables S2**.

### Protein levels distinguished malaria cases *vs*. controls

**Figure 3:**
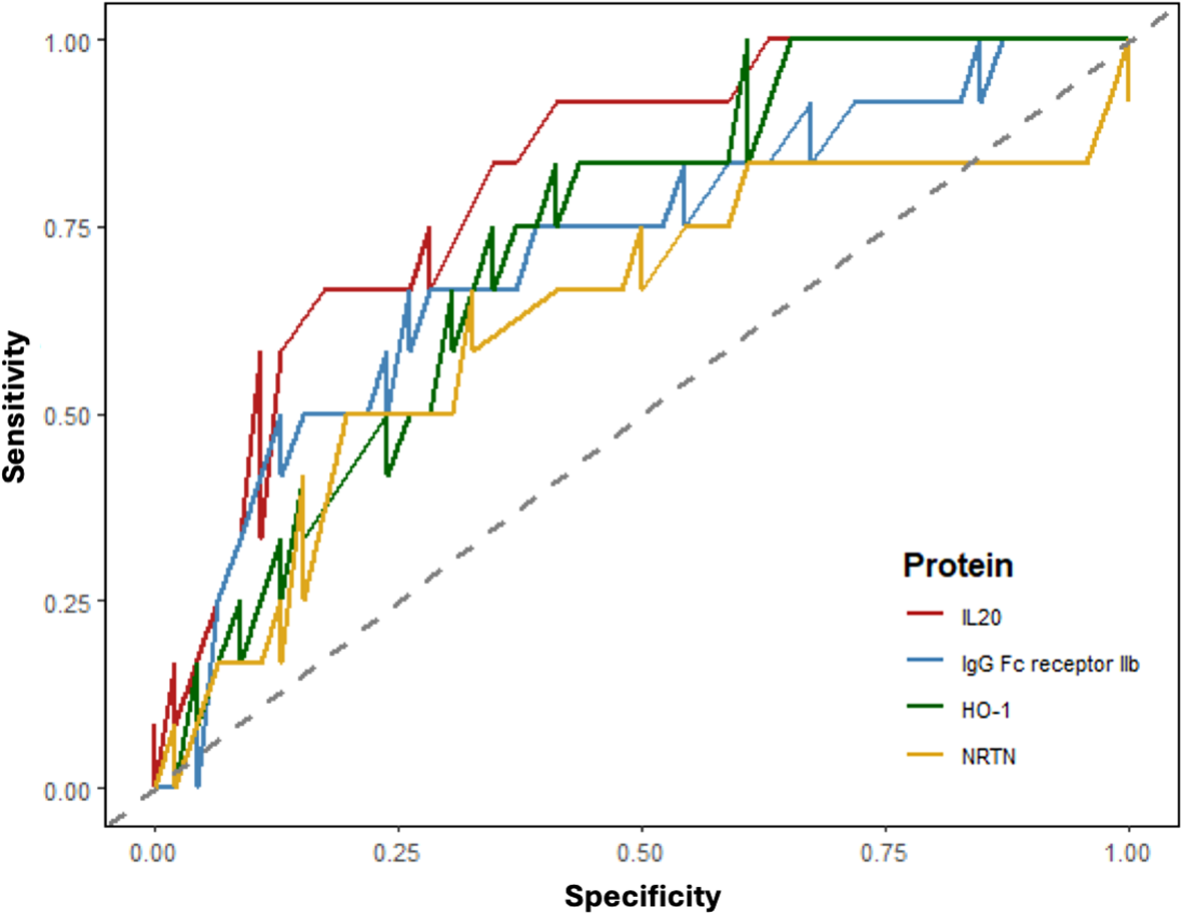
ROC curves for different protein expression levels in malaria positive *vs.* malaria negative women. Each protein has been assigned a color code, as indicated in the legend. Dashed diagonal line represents the line of no discrimination (AUC =0.5).

We furthermore assessed whether a combination of plasma proteins can distinguish malaria cases *vs.* controls. This analysis revealed varying predictive performance among the four proteins we examined in relation to malaria status. IL-20 protein exhibited the highest discriminatory power (AUC = 0.815), indicating a strong association with malaria status (**Figure 3**). This suggests that IL-20 expression levels serve as a promising biomarker for malaria diagnosis or prognosis. FCGR2B protein demonstrated moderate discriminatory ability (AUC = 0.717), suggesting a notable but less pronounced association with malaria status compared to IL-20. Similarly, HO-1 protein also displayed moderate discriminatory power (AUC = 0.729), indicating some association with malaria status, although not as strong as IL-20. NRTN (Q99748) protein, with the lowest AUC value (AUC = 0.635), showed weaker discriminatory ability compared to the other proteins. Although NRTN expression levels may still have some association with malaria status, the association is less pronounced compared to IL-20, FCGR2B, and HO-1.

## Discussion

**Figure 4:**
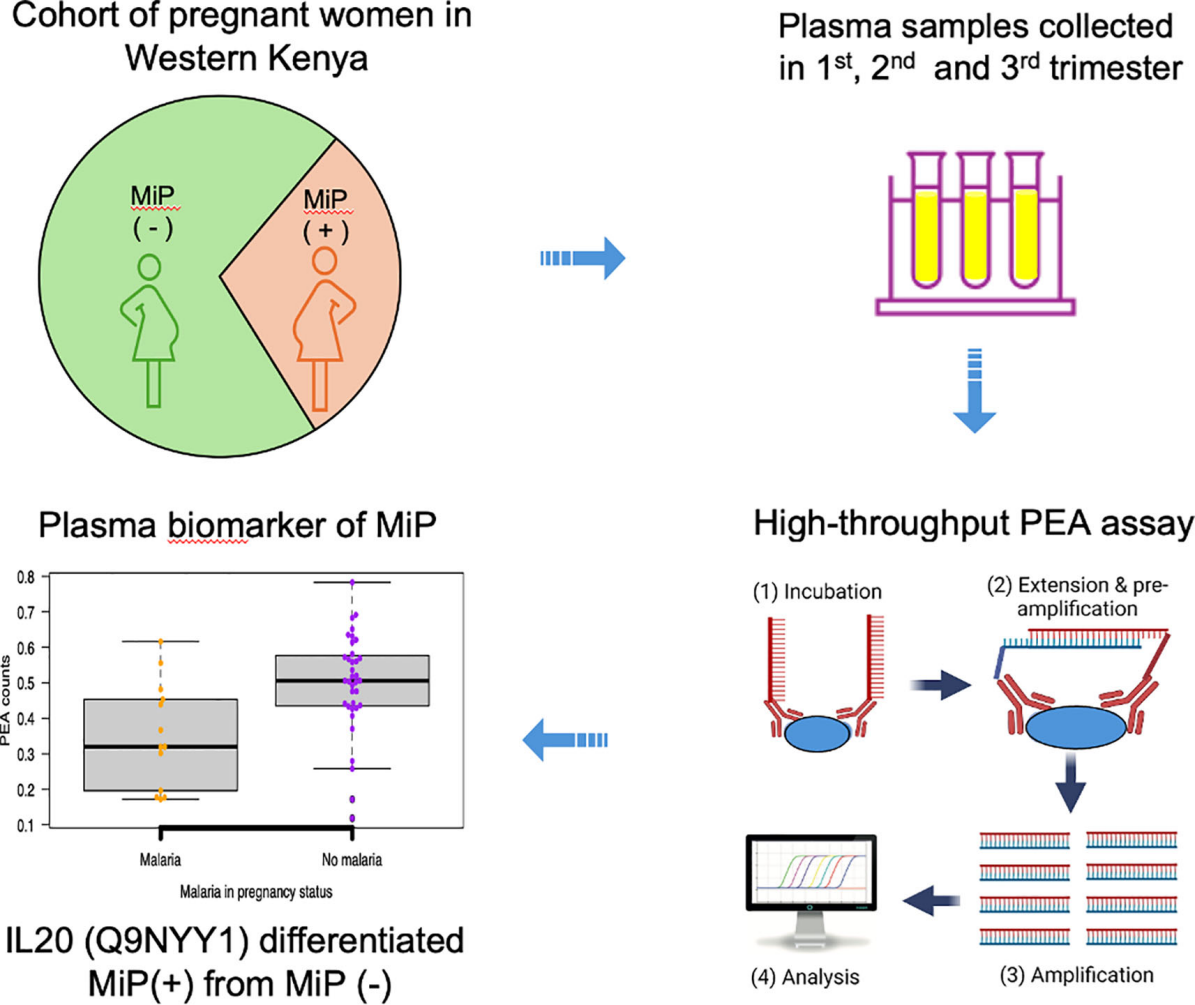
This figure summarizes the approach applied to identify plasma biomarkers of malaria during pregnancy leveraging a high-throughput multiplex PEA, that utilizes minute amount of blood sample.

Accurate tools for diagnosing malaria during pregnancy are important, but the greater clinical need lies in identifying markers that can predict placental dysfunction or adverse outcomes among *P. falciparum*-infected pregnant women. This study represents a discovery-phase effort focused on identifying plasma proteins associated with *P. falciparum* infection in pregnancy, forming a foundation for future validation studies that explore their relevance to pregnancy outcomes (Friedman et al., 2010). In this study, we aimed to identify potential markers of malaria infection by using high-throughput PEA technology (summarized in **Figure 4**). We observed that FCGR2B, HO-1, NRTN and IL-20 were differentially expressed in plasma from malaria-infected pregnant women *vs.* from non-malaria-infected controls. Moreover, these proteins may also highlight processes that underlie malaria pathophysiology during pregnancy.

FCGR2B, a low affinity immunoglobulin gamma Fc region receptor, has multiple isoforms in macrophages, lymphocytes, and IgG-transporting placental epithelium (Ding et al., 2023). It is the only inhibitory Fc receptor that controls many aspects of immune and inflammatory responses (Stuart et al., 1989). Variations in the gene encoding this protein, such as loss-of-function polymorphism, have implications for autoimmunity and infection, including severe malaria. FCGR2B is involved in several processes, including Fc receptor-mediated immune complex endocytosis, nervous system development, and immune responses (Smith and Clatworthy, 2014). Other studies have identified FCGR2B as a marker of human metastatic melanoma, where its expression impairs the tumor susceptibility to FcgammaR-dependent innate effector responses (Bournazos et al., 2009). With the availability of monoclonal antibodies against FCGR2B (Cassard et al., 2008), studies are needed to evaluate its potential as a biomarker of PAM.

We also identified HO-1 as abundantly expressed in PAM cases. HO-1 plays a crucial role in heme degradation and cellular responses to stress (Veri et al., 2007). During pregnancy, HO-1 maintains a balanced immune response and supports healthy placental development (Ozen et al., 2021). Evidence from a mouse model (Ozen et al., 2021) indicates that it helps protect against oxidative stress and inflammation, which are often elevated in pregnancy-associated conditions. We hypothesize that in the context of pregnancy-associated malaria, HO-1 may play a role in mitigating the adverse effects of the infection by reducing oxidative stress and inflammation.

Two other proteins that decreased in PAM cases, NRTN and IL-20, deserve further evaluation. NRTN has been implicated in supporting healthy placental growth and development. It is also involved in the regulation of blood vessel formation and trophoblast invasion, which are critical for successful pregnancy outcomes (Schumacher and Zenclussen, 2015). IL-20 plays a critical role in the development and maintenance of the placenta, as well as in the regulation of trophoblast invasion, angiogenesis and vascular remodeling (Morel et al., 2020). Thus, IL-20 downregulation during PAM might contribute to poor pregnancy outcomes. Indeed, disruptions in IL-20 signaling have been associated with adverse pregnancy outcomes (Menon et al., 2006). In the context of pregnancy-associated malaria, IL-20 may modulate the inflammatory response and placental immune tolerance, potentially impacting the severity and outcomes of the infection. These proteins need further evaluation.

Our findings align with and expand upon previous research exploring biomarkers of *P. falciparum* infection in pregnancy which highlighted the role of inflammatory cytokines and immune dysregulation in PAM (Boström et al., 2012; Megnekou et al., 2015). Ruizendaal et al. demonstrated altered angiogenic marker profiles in infected women, consistent with observations of differential expression of IL-20, a cytokine implicated in vascular remodeling (Ruizendaal et al., 2017). These insights reinforce the idea that PAM involves complex immune-endothelial interactions, and that panels like those used in our PEA approach are well-suited to uncover such proteomics changes. By integrating high-throughput proteomics and rigorous statistical modeling, we corroborate these prior reports of immune dysregulation in PAM (Boström et al., 2012; Megnekou et al., 2015) but extend these findings by identifying FCGR2B and IL-20 as novel candidates linked to placental sequestration. These markers could complement previously identified candidates and enhance the diagnostics landscape for PAM.

The choice of two Olink panels, Inflammation and Cardiovascular II, provided a wide repertoire of proteins with a critical role during pregnancy or with diagnostic capability for assessing malaria infection of the placenta. However, the panels could have excluded other potentially important proteomic targets, and future studies should include placental-specific proteins, e.g. placental alkaline phosphatase, to improve biomarker coverage. This study involved a relatively small sample size and was based on a single study center. Hence, these observations warrant further validation using larger, multicenter malaria-in-pregnancy cohorts. Because this study is correlative, future studies will also seek to experimentally validate the involvement of the identified factors in malaria during pregnancy and the underlying mechanisms, which may uncover additional diagnostic and therapeutic strategies.

In conclusion, this study identified four proteins that were differentially regulated between malaria positive and malaria negative infected women. While the study does not establish a direct link between the identified protein markers and clinical outcomes such as placental infection, low birth weight, or preterm birth, it highlights a distinct panel of proteins that are differentially expressed in *P. falciparum*-infected pregnant women. We, therefore, propose IL-20 and FCGR2B as lead biomarkers for PAM, with validation in outcome-linked cohorts needed to assess their utility in predicting placental dysfunction, e.g. low birth weight and preterm delivery.

## Data Availability

The raw data supporting the conclusions of this article will be made available by the authors, without undue reservation.
